# Phytoestrogens in Soy Infant Formula: Association with DNA Methylation in Girls Has Unknown Implications

**DOI:** 10.1289/ehp.125-A61

**Published:** 2017-03-01

**Authors:** Nate Seltenrich

**Affiliations:** Nate Seltenrich covers science and the environment from Petaluma, CA. His work has appeared in *High Country News*, *Sierra*, *Yale Environment 360*, *Earth Island Journal*, and other regional and national publications.

For years, parents have contended with conflicting reports^1^ ^,^ ^2^ ^,^ ^3^ ^,^ ^4^ ^,^ ^5^ in the media and blogosphere on the safety of soy infant formula. Soybeans contain phytoestrogens, which under some conditions mimic or interfere with the estrogens within the human body.^6^ However, the National Toxicology Program concluded in 2009, based on the research to that point, that exposures to phytoestrogens in soy formula are of “minimal concern.”^7^ Research in the field continues apace, with a new study providing evidence of an association between soy formula consumption and differences in gene methylation in baby girls—although any health implications remain unknown.^8^

**Figure:**
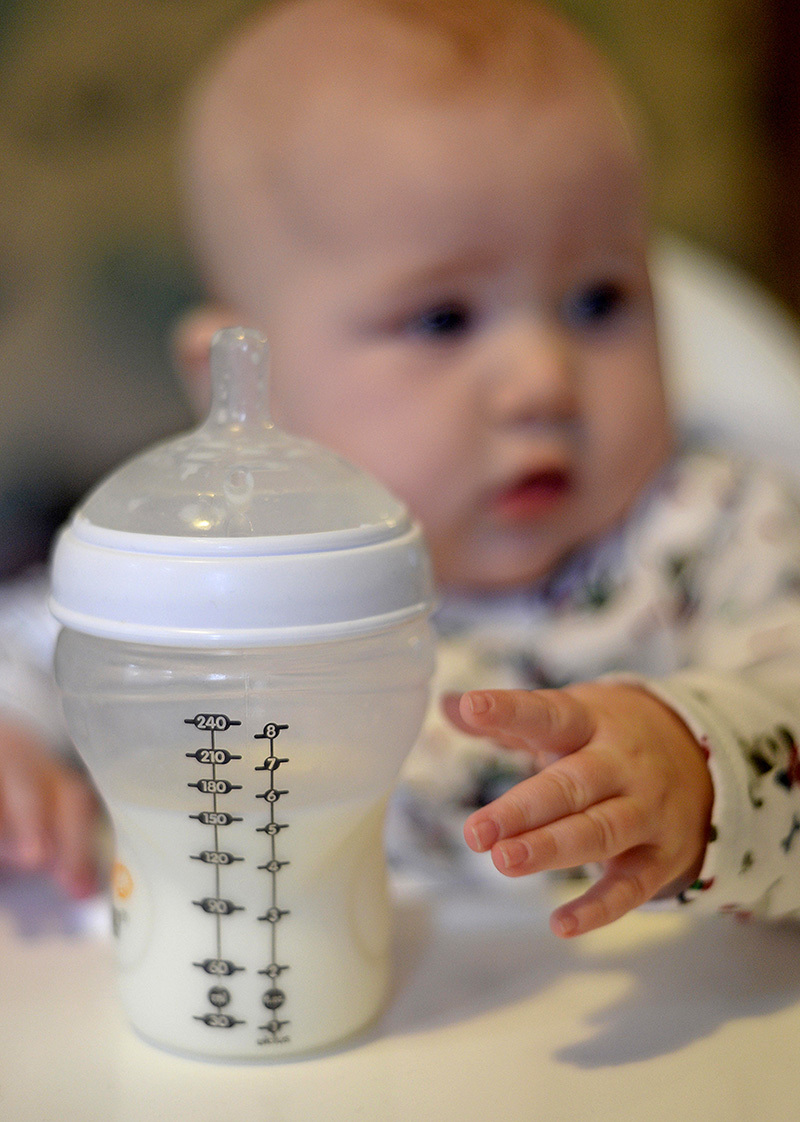
A new analysis suggests that specific methylation patterns may result from consuming soy formula, although it is unclear whether the changes would be relevant to babies’ health. © PA Images/Alamy Stock Photo

The research is part of the larger Infant Feeding and Early Development (IFED) study, launched in 2010 to identify differences in estrogen-responsive outcomes among infants fed cow formula, soy formula, or breast milk.^9^ Researchers collected vaginal epithelial cells from baby girls and submitted them for epigenome-wide analysis. This analysis identified a specific gene, *PRR5L*, that showed differences in methylation between baby girls fed soy formula during their first 9 months of life and those fed cow formula.^8^ The role of *PRR5L* within the context of vaginal epithelial cells is unknown.

The researchers tested the findings of their methylation analysis through an animal study in which they injected mice with a purified form of genistein, the predominant phytoestrogen in soy formula. The dose they used was intended to produce serum concentrations comparable to those in formula-fed infants. They found that dosed mice had lower expression of *Prr5l* (the mouse version of *PRR5L*) than controls.^8^

DNA methylation is a healthy, vital process that can vary in response to conditions that change over time, but certain differences have been linked with a range of human diseases.^10^ It is unclear whether the differences in methylation seen in this study have any clinical relevance, and in any case the study was not intended to explore health outcomes. The researchers also cannot say whether the observed differences are persistent or fleeting.

“We don’t interpret [the findings] as a contraindication for soy formula use for those kids in whom it is specifically indicated,” says senior author Jack Taylor, a principal investigator at the National Institute of Environmental Health Sciences. “We’re being intentionally very cautious. This is one study, and it shows an effect that we think is interesting and useful for the scientific community to know about.”

Despite the recommendation by the American Academy of Pediatrics (AAP) that babies be exclusively breastfed during their first 6 months of life,^11^ by 2 months most infants in North America have begun receiving at least some formula.^12^ Most parents turn to cow formula, but soy formula has been an alternative for babies who are allergic to cow’s milk or whose households follow a vegan diet.

Today soy formula represents an estimated 12% of the U.S. formula market,^7^ a share that by all accounts is on the decline. This trend is due at least in part to the AAP’s latest published guidance on the subject,^12^ which states that there are few indications for the use of soy formula in place of cow formula, even in cases of allergy. But as numerous prominent websites and blogs suggest, another likely component in the decline in sales is consumer concern over potential adverse health effects of soy formula, especially in regards to estrogenic activity.

With respect to the current study, the animal model is a source of significant concern for researcher Thomas Badger of the University of Arkansas for Medical Sciences, who was not involved in the study. “No baby in the world would ever be subjected to a subcutaneous injection of purified genistein,” he says. “It’s not modeling what happened with the babies fed soy formula as discussed in the first part of the [research article]. It’s completely different.”

Instead, Badger suggests that future studies use an animal model with an endocrine system and developmental profile similar to that of humans, such as monkeys or pigs, so that they can be fed similarly to human infants. “Once you see changes, you can ask questions about them,” he says. His own research using such an approach in pigs has not identified any significant adverse developmental effects of soy formula, and in fact suggests some benefits.^13^ ^,^ ^14^

Researcher Heather Patisaul of North Carolina State University says the findings warrant further research into potential effects of soy formula on female reproductive health. “The obvious next step is to see if changes in this gene are biologically meaningful in any way,” she says.
